# The Short QTc Is a Marker for the Development of Atrial Flutter and Atrial Fibrillation

**DOI:** 10.1155/2020/2858149

**Published:** 2020-11-08

**Authors:** Simon W. Rabkin, Jacky K. K. Tang

**Affiliations:** Division of Cardiology, University of British Columbia, Vancouver, Canada

## Abstract

A short QT interval has been difficult to define, and there is debate whether it exists outside of an extremely small group of individuals with inherited channelopathies and whether it predicts cardiac arrhythmias. The objective was to identify cases with short QT and their consequences. Our hospital ECG database was screened for cases with a QTc based on the Bazett formula (QTcBZT) of less than 340 ms. The QTc was recalculated using the spline (QTcRBK) formula, which more accurately adjusts for the heart rate and identifies cases based on percentile distribution of the QT interval. The exclusion criteria were presence of bundle branch block, arrhythmias, or electronic pacemakers. An age- and sex-matched cohort was obtained from individuals with normal QT intervals with the same exclusion criteria. There were 28 cases with a short QTc (QTcRBK < 380 ms). The age was 69.6 ± 14.6 years (mean ± SD) (50% males). The QT interval was 305.7 ± 61.1 ms with QTcRBK 308.4 ± 31.4 ms. Subsequent ECGs showed atrial flutter in 21%, atrial fibrillation in 18%, and atrial tachycardia in 4% of cases. Thus, atrial arrhythmias occurred in 43% of cases. This incidence was significantly (*p* < 0.0001) greater than the incidence of atrial arrhythmias in age- and sex-matched controls. In conclusion, a short QT interval can be readily identified based on the first percentile of the new QTc formula. A short QTc is an important marker for the development of atrial arrhythmias, including atrial flutter and atrial fibrillation, with the former predominating. It should be part of patient assessment and warrants consideration to develop strategies for detection and prevention of atrial arrhythmias.

## 1. Introduction

A short QT interval has been difficult to define, and there is debate whether it exists outside of an extremely small group of individuals with inherited channelopathies and whether it predicts cardiac arrhythmias. There is no doubt that genetic mutations in cardiac ion channels can be associated with short QT intervals [1–6]. Furthermore, these familial cases with mutations in ion channels are associated with ventricular arrhythmias, atrial fibrillation, and sudden death [7, 8]. Most of these cases have been identified in young individuals consistent with a genetic alteration in cardiac ion channels [2, 3]. There has been controversy as to whether the short QT interval is a relevant entity in the general population. In the general population, it is an extremely uncommon entity [9–12].

The implication of the presence of a short QT interval, apart from the rare genetic conditions, has been examined from the perspective of either total mortality, or fatal arrhythmias, or atrial fibrillation. Some studies that examined mortality did not capture data on cardiac arrhythmias or focused only on ventricular arrhythmias [9]. Nielsen et al. examined the 12-lead electrocardiogram (ECG) of 281,277 individuals in Copenhagen who were followed up for an average of 5.7 years. They reported an increased risk of atrial fibrillation (a hazard ratio of 1.68) for individuals with a short QTc [13]. In contrast, Mandyam et al. assessed the relationship between the QT interval and the incidence of atrial fibrillation in three cohorts—the Atherosclerosis Risk in Communities (ARIC) cohort (*n* = 15,792), the Cardiovascular Health Study (CHS) cohort (*n* = 5,888), and the Health, Aging, and Body Composition (Health ABC) study cohort (*n* = 3,075) [14]. They did not find a consistent relationship between a short QTc and the incidence of atrial fibrillation [14]. The association of short QT intervals with cardiac arrhythmias in the general population is dependent on the definition of the short QT interval, which in turn is influenced by ensuring a correct adjustment for the well-known inverse association between QT duration and heart rate. Some authorities suggest that an absolute value of QT may be used for the definition of short QT [15]. The Bazett formula is used by many studies and guidelines to define the short QTc [10, 16, 17]. Unfortunately, this formula is only optimal in cases with a heart rate of around 60 bpm and becomes progressively less accurate at faster and slower heart rates [18–20]. Recently, a new QT-heart rate correction formula was developed based on the ECGs from about 13,600 individuals in the United States' National Health and Nutrition Examination Survey (NHANES) population study and was shown to be relatively independent of heart rate and also superior to other formulae [21].

The short QT interval has also been related to early repolarization changes [22, 23]. In turn, early repolarization changes have been related to adverse outcomes [24], although this is controversial [25, 26]. Early repolarization changes can be transient and are impacted by the activity and heart rate [27], and as such, it is important to utilize an appropriate heart rate correction formula to ensure that the short QT is defined appropriately.

The objective of the present study was to examine the short QT interval by applying the recently developed QTc spline heart-rate correction formula, and to determine whether it is associated with early repolarization (ER) changes and/or cardiac arrhythmias.

## 2. Methods

Our hospital ECG database was screened for cases with a computerized calculation of QTc based on the Bazett formula (QTcBZT) of less than 340 ms. This value was selected because it was recommended in the 2015 European Society of Cardiology guidelines for the diagnosis of the short QT syndrome [28]. In the case where family history of sudden death or genetic testing information is not available, such as in our study, a QTc ≤ 340 ms was recommended for the diagnosis of short QT syndrome and was given a Class I and Level C recommendation [28]. The abbreviation nomenclature for QT-heart rate correction formulae used standardized abbreviations [20]. The study was approved by our institution's ethics committee for evaluation of ECGs and age and sex data. All 12-lead electrocardiograms were recorded digitally on Muse™, version 9.0 SP6 (General Electric, Boston, USA). The exclusion criteria were presence of bundle branch block, arrhythmias, or electronic pacemakers. ECGs were then reviewed by a cardiologist (SWR). The ECGs were viewed on a 10-second rhythm strip mode at 25 mm/s paper speed. Cases were excluded when ST-T changes precluded the accurate measurement of QT intervals, or the RR interval or QT interval was erroneously measured by computer calculations. Digital calipers were used to measure two to three RR intervals, and the corresponding QRS durations and QT intervals were measured. The heart rate calculations for the selected cases were verified by manual measurements utilizing the software's digital calipers.

The initial screen for short QT cases used the Muse system (Muse™, version 9.0 SP6; General Electric, Boston, USA) in which the QT interval is measured “from the earliest detection of depolarization in any lead (QRS onset) to the latest detection of repolarization in any lead (T offset) (ECG Analysis Program; GE Healthcare Milwaukee, WI, United States). The QT interval and heart rate measured by the analysis program were used in the initial screening heart rate adjustment formulae. The ECGs were then examined in detail. The QT interval was measured from the initial deflection of the QRS complex to the end of the T-wave. All 12 leads were visually inspected for the lead with the most prominent deflections, which was then used to make the measurements using digital calipers. The end of the *T*-wave was identified (using the tangent method) as the intersection of a tangent drawn to the steepest last limb of the *T*-wave with the baseline as outlined in detail elsewhere [29]. The QT interval was measured from the lead with the longest QT interval of all leads, usually V2, according to guidelines [30].

The ECGs were reviewed for the presence of early repolarization (ER) changes. MacFarlane's consensus criteria for ER were used [31] and are briefly as follows: (i) an end-QRS notch or slur on the final 50% of the down-slope of an *R* wave in which the notch or the slur was above the baseline, (ii) Jp ≥ 0.1 mV in 2 or more contiguous leads of the 12-lead ECG (excluding V1–V3; to eliminate the possibility of Brugada syndrome), and (iii) QRS duration < 120 ms, as per our previous examination of early repolarization changes [27].

An age- and sex-matched cohort was obtained from the same ECG database for individuals with normal QT intervals (QTcBZT 341–450 ms for men and 342–460 ms for women). The exclusion criteria were presence of bundle branch block, arrhythmias, or electronic pacemakers. A list of controls was randomly generated 2 : 1 age- and sex-matched ECGs by the computer algorithm on Muse™, version 9.0 SP6 (General Electric, Boston), for the sinus rhythm and normal QTc. As many of the original short QT ECGs were excluded (because of the exclusion criteria), there was an excess of controls available. Age- and sex-matched individual ECGs were then examined in the order in which they were generated by the software. Cases with arrhythmia, or ST-T changes precluding accurate QT measurement, were excluded. ECGs were selected (or excluded) as per the above criteria until a 2 : 1 ratio of controls to cases was achieved. The 2 : 1 age and sex match was then verified. Subsequently, all ECGs within two months following the index ECG were reviewed for the presence of any arrhythmias.

A new QT-heart rate correction formula (QTcRBK) has been developed utilizing the ECGs from approximately 13,600 individuals in the United States' National Health and Nutrition Examination Survey (NHANES) population study and has been shown to be relatively independent of heart rate and also superior to other formulae [21]. We used the nomenclature for QTc abbreviations that specified the first three syllables of the first author's name [20]. Based on the new spline formula (QTcRBK), a short QTc is defined at the first percentile in that population and is less than 380 ms in both men and women [32].

The data are presented as the mean±1 SD. A chi-square test was used to examine the occurrence of atrial arrhythmias in cases with short QT compared with controls.

## 3. Results

There were 28 cases with a short QTc (QTcRBK < 380 ms). The age was 69.6  ±14.6 years (mean ± SD) (50% males). None of the cases of short QTc had ST changes that fulfilled the criteria for early repolarization changes [31].

**Figure 1:**
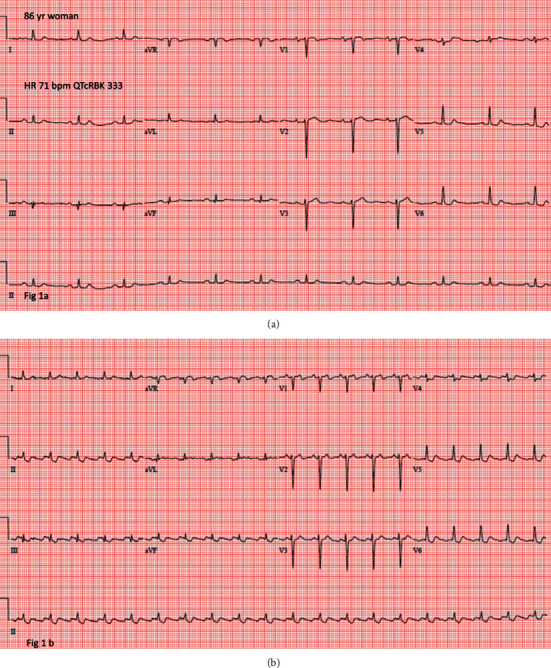
ECG of an individual with a short QT interval (a) and the subsequent development of atrial flutter (b).

An illustrative case is an 86-year-old woman with a short QT interval (Figure 1(a)) who subsequently developed atrial flutter (Figure 1(b)).

**Figure 2:**
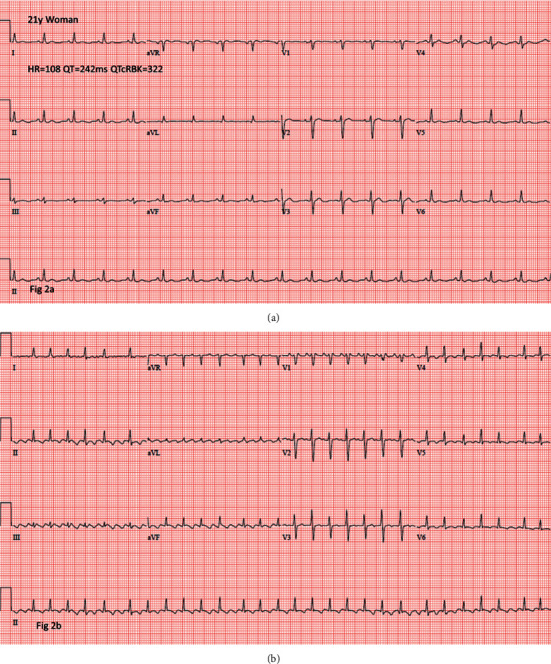
ECG of an individual with a short QT interval (a) and the subsequent development of atrial flutter (b).

**Figure 3:**
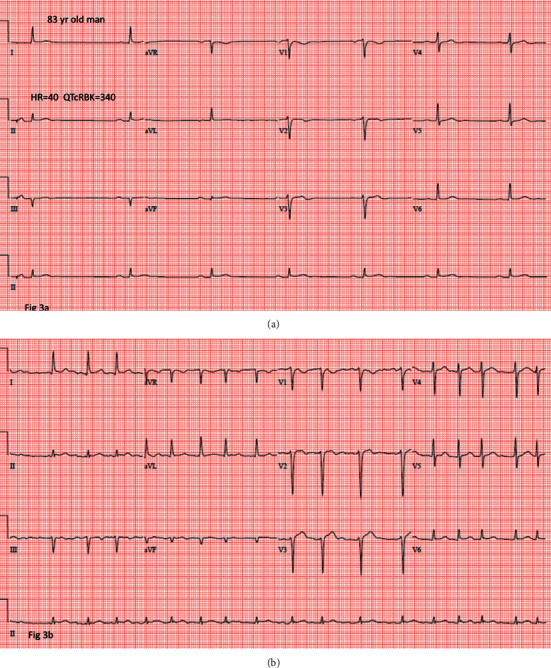
ECG of an individual with a short QT interval (a) and the subsequent development of atrial fibrillation (b).

Another example is a 21-year-old woman with a short QT interval (Figure 2(a)) who also developed atrial flutter (Figure 2(b)). Atrial fibrillation also occurred, which is illustrated by an 83-year-old man with a short QT interval (Figure 3(a)) who developed atrial fibrillation (Figure 3(b)).

**Figure 4:**
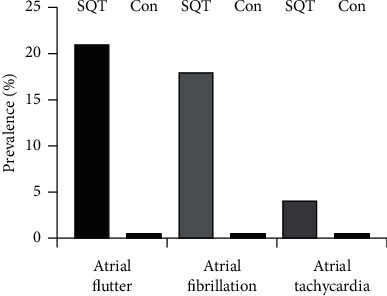
Prevalence of atrial flutter, atrial fibrillation, and atrial tachycardia in patients with a short QT interval compared with the control group with a normal QT interval (see text for methodology for selection of control cases).

The short QT was associated with the development of atrial flutter in 21%, atrial fibrillation in 18%, and atrial tachycardia in 4% of cases (Figure 4). Thus, atrial arrhythmias occurred in 43% of cases. In order to perform a case-control study, an age- and sex-matched cohort was obtained by randomly generating 2 : 1 age- and sex-matched ECGs using a computer algorithm from individuals with normal QT intervals using the same database. All ECGs within two months following the index ECG were reviewed for the presence of any arrhythmias. The control group had the same age as the short QT group (69.6  ±14.5 vs 69.6 ± 14.6 years, respectively) and the same ratio of men and women (1 : 1). This is an indicator of the goodness of fit of the control group. The short QT group had a mean heart rate of 71.3 ± 22.3 bpm and a QTc of 305.7 ± 61.1 ms, QTcBZT of 319.5 ± 25.3 ms, and QTcRBK of 308.4  ±31.4 ms. In the control group, the heart rate was 75.8 ± 15.3 bpm, and the QTc was 384.1 ± 33.0, QTcBZT 426.7± 21.3, and QTcRBK 406.7 ± 18.0 ms.

There were no recorded cardiac arrhythmias in the control group. The occurrence of atrial arrhythmias in the short QTc group was significantly (*p* < 0.0001) greater than in the control group in which no recorded subsequent atrial rhythms were identified.

## 4. Discussion

The major contributions of the study are the demonstration of high prevalence of atrial arrhythmias in patients with short QT intervals when the heart rate is adjusted by a new QTc formula. Especially noteworthy is the high prevalence of atrial flutter, which has not been previously recognized to be associated with the short QTc. The results utilized a rigorous case-control methodology to conclude that there was a significant increase in atrial arrhythmia frequency in this population.

Our population had a mean age of 70 years. This is consistent with other studies. Iribarren et al. reported a monotonic increase in the prevalence of short QT with advancing age [9], while Miyamot et al. found a biphasic age distribution with a peak at older as well as younger ages [17]. As our database is from an adult general hospital, there would not be people at a young age. Our population had an equal number of men and women, which was similar to the data in the Kaiser Permanente study [9]. Considering the age of our patient population, it is highly unlikely that they had monogenic short QT syndrome that would have been manifest in childhood. We hypothesize an aging-induced alteration in channel functionality and further hypothesize that such a change is connected to the known aging-associated increase in the occurrence of atrial arrhythmias. An alternative hypothesis is that single-nucleotide polymorphisms in relevant channel genes only manifest at older ages.

Potential issues with regard to QT measurement in the short QT situation merit discussion. The tangent method for determining the end of the *T* wave relies on the slope of the descending portion of the *T* wave, which can be steep in leads such as V2 in some cases especially with *T* waves with an increased magnitude suggesting that this method might produce shorter QT than other approaches such as the threshold- or area-based methods. It is unlikely that the method of QT measurement affected our results as the screening approach to the selection of cases and controls used the computerized assessment of “the earliest detection of depolarization in any lead (QRS onset) to the latest detection of repolarization in any lead (T offset)” (Muse™, version 9.0 SP6). We then analyzed the cases in detail in order to ensure that the QT interval was in the 1% of the normal population [32].

QTc is a measurement of ventricular repolarization, and atrial depolarization is not readily apparent on the 12-lead ECG. Repolarization in the two different tissues can be subject to the same factors. For example, selective inhibition of cardiac late inward sodium reduces both atrial and ventricular repolarization [33, 34]. The *α*-subunit of the myocardial *I*_*Kr*_-channel, encoded by the KCNH2 gene, regulates both ventricular and atrial repolarization [35] Patients with mutations in KCNH2 have a higher incidence of atrial fibrillation, although the change in ventricular repolarization may be more evident [35]. Thus, the QTc can be a window on atrial repolarization.

Our cases of atrial flutter fulfilled the definition, with the presence of flutter waves with a biphasic sawtooth-like oscillations at a rate between 200 and 400 bpm [36]. The intriguing and unanswerable question is whether they represented a subtype of atrial flutter that might eventually transition into atrial fibrillation in the future [36].

The QT interval on the surface ECG is an approximation of cellular-level events, specifically the duration of ventricular transmembrane action potentials, but it is more complex as it includes cell-to-cell communication as well as the net effect of these forces reaching the body surface [37]. Nevertheless, correlations with the cellular events can provide different levels of understanding, recognizing that our study population is unlikely due to a genetic mutation. The molecular mechanisms of the short QT in genetic mutations include gain of function in potassium channels (KCNH2, KCNQ1, and KCNJ2) and loss of function mutations in *L*-type calcium channels (CACNA1C and CACNB2b) [1–6, 38]. The clinical setting of the patients in this study may represent states of increased function of potassium channels or inhibition of *L*-type calcium channels. Secondary causes of the short QT interval include situations of hyperkalemia, hypercalcemia, acidosis, and hyperthermia [15]. These conditions, however, are rarely associated with arrhythmias such as atrial flutter [39, 40], with the rare exception of an associated mutation in sodium channels [41].

The genetic conditions associated with short QT intervals have been found to be gain of function mutations in the late potassium channels leading to abbreviating the action potential and refractoriness that are posited to enhance re-entry mechanisms leading to atrial fibrillation, as well as ventricular fibrillation [15].

There are a number of insights from human-induced pluripotent stem cell models of the short QT syndrome (SQTS) derived from an individual with SQTS type-1 with the N588K mutation in the KCNH2 gene [42]. This gene defect is not only associated with a shortened action potential duration, impaired action potential duration-rate adaptation, and abbreviated refractory period but also augmented *I*_*Kr*_ current due attenuated inactivation of *I*_*Kr*_ and increased expression of its channel protein [42]. Networks of these cells not only manifest an abbreviated wavelength of excitation but also increased inducibility of sustained spiral waves, accelerated with stabilized rotors manifested by increased rotor rotation frequency, curvature, and complexity [42]. Using a protocol of systematic electrical programmed simulation, sustained re-entrant arrhythmias were readily induced in SQTS preparations, which were highly stable and persistent [42]. The similarity between the cellular model of SQTS and rotors operative in atrial fibrillation [43] strengthens the connection between SQTS and atrial fibrillation. With the caveats that the stem cell model of short QT was a specific type of inherited SQTS and is a specific cellular model [42], it nevertheless raises the question that factors leading to increased I_Kr_ in the clinical setting not only produce a short AP which is manifested as a short QTc but also predispose to the ready induction of rotors which underlie atrial arrhythmias.

There have been reports of short QT intervals associated with early repolarization changes [22, 23]. We did not find a single case in our population. This may reflect the older age group of the cases or the dynamic nature of early repolarization changes. We have previously reported that repolarization changes are more evident at slow heart rates and less evident at faster heart rates [27]. The majority of the cases in this study did not have bradycardia.

## 5. Limitations

There are a number of factors that need to be considered in interpretation of the results. First, this is a retrospective case-control study, which carries all the limitations and potential biases associated with this kind of study. There are, however, circumstances in which this kind of study is useful and necessary, specifically in cases of infrequent conditions such as the short QT syndrome. Many of the studies in this field are retrospective case-control studies because of the infrequency of the short QT. Second, there are a number of medical conditions of the patients in the study that might have been responsible for the short QT interval. A detailed examination of each case was beyond the (approved) scope of this study. Third, and importantly, we had no control over the length of observation in the hospital and the number of ECGs done so that there might easily have been discrepancies between the short QT and the control group with respect to follow-up and ECGs. Fourth, other factors such as catecholamine excess may have been responsible for the short QT interval and cardiac arrhythmias. If this was the case, then the short QT would still be of value as it would be a marker for the causative factor. Fifth, the number of cases is small. However, this should be expected considering our definition of short QT was individuals in the first percentile of the QTc distribution.

## 6. Conclusion

The study identifies, in essentially a hospital-based population, the association of the short QT interval and atrial arrhythmia. The strong association with atrial flutter is noteworthy. The study demonstrates the value of utilizing a more accurate heart rate correction formula, especially in the clinical setting of faster heart rates as it identifies individuals with a shorter QTc. Evaluation for the presence of a short QTc should be part of patient assessment and warrants consideration to develop strategies for the prediction and prevention of atrial arrhythmias.

## Data Availability

The data used to support the findings of this study are not available, as there are personal identifiers and our Ethics Committee has not allowed this to be done.
